# Intelligent Sensor Positioning and Orientation Through Constructive Neural Network-Embedded INS/GPS Integration Algorithms

**DOI:** 10.3390/s101009252

**Published:** 2010-10-15

**Authors:** Kai-Wei Chiang, Hsiu-Wen Chang

**Affiliations:** Department of Geomatics, National Cheng-Kung University, No.1, Ta-Hsueh Road, Tainan 701, Taiwan; E-Mail: kwchiang@mail.ncku.edu.tw

**Keywords:** GPS/INS, sensor integration, mobile mapping systems, constructive neural networks

## Abstract

Mobile mapping systems have been widely applied for acquiring spatial information in applications such as spatial information systems and 3D city models. Nowadays the most common technologies used for positioning and orientation of a mobile mapping system include a Global Positioning System (GPS) as the major positioning sensor and an Inertial Navigation System (INS) as the major orientation sensor. In the classical approach, the limitations of the Kalman Filter (KF) method and the overall price of multi-sensor systems have limited the popularization of most land-based mobile mapping applications. Although intelligent sensor positioning and orientation schemes consisting of Multi-layer Feed-forward Neural Networks (MFNNs), one of the most famous Artificial Neural Networks (ANNs), and KF/smoothers, have been proposed in order to enhance the performance of low cost Micro Electro Mechanical System (MEMS) INS/GPS integrated systems, the automation of the MFNN applied has not proven as easy as initially expected. Therefore, this study not only addresses the problems of insufficient automation in the conventional methodology that has been applied in MFNN-KF/smoother algorithms for INS/GPS integrated systems proposed in previous studies, but also exploits and analyzes the idea of developing alternative intelligent sensor positioning and orientation schemes that integrate various sensors in more automatic ways. The proposed schemes are implemented using one of the most famous constructive neural networks—the Cascade Correlation Neural Network (CCNNs)—to overcome the limitations of conventional techniques based on KF/smoother algorithms as well as previously developed MFNN-smoother schemes. The CCNNs applied also have the advantage of a more flexible topology compared to MFNNs. Based on the experimental data utilized the preliminary results presented in this article illustrate the effectiveness of the proposed schemes compared to smoother algorithms as well as the MFNN-smoother schemes.

## 1. Introduction

The development of land based mobile mapping systems was initiated by two research groups in North America, The Center for Mapping at Ohio State University, USA, and the Department of Geomatics Engineering at the University of Calgary, Canada [1,2]. Starting in the early 2000s, a number of land based mobile mapping systems have been utilized in commercial applications [2–9]. The process of mobile mapping is basically executed by producing more than one image that includes the same object from different positions, and then the 3D positions of the same object with respect to the camera frame can be measured [1].

**Figure 1.**
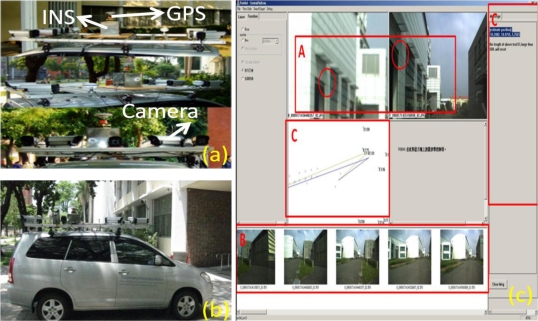
An example of a land based mobile mapping system.

Since the early nineties, advances in satellite and inertial technology made it possible to think about mobile mapping in a new way. Instead of using ground control points as references for orienting the images in space, the trajectory and orientation of the imager platform can now be determined directly [3]. Cameras, along with positioning and orientation sensors, are integrated and mounted on a land vehicle for mapping purposes. Objects of interest can be directly measured and mapped from images that have been georeferenced using positioning and orientation sensors [10]. An example of land based mobile mapping system is illustrated in Figure 1.

Figure 1a gives an overall view of the sensors onboard the land based mobile mapping system from different perspectives, and Figure 1b depicts an overall view of the mobile mapping van. In addition, Figure 1c illustrates an example of direct geo-referencing the corner of interest shown in green dots from two geo-referenced images. This procedure is accomplished through the use of precise positioning and orientation techniques.

An INS is a self-contained navigation technique in which measurements provided by accelerometers and gyroscopes are used to track the position and orientation of an object relative to a known starting point, orientation and velocity [11]. In addition, GPS is a universal, all-weather, world-wide positioning system that provides time, position and velocity data. Both systems can be used as stand-alone navigation tools or in conjunction with other sensors for various purposes. Moreover, the integration of GPS and INS can overcome problems with environments like urban canyons, forests and indoor settings where GPS alone cannot provide service; for more information see [12–15]. In order to attain reasonable accuracies of position and orientation solutions, a tactical grade or higher quality INS along with GPS has been applied as the primary position and orientation system for current commercial systems [1]. However, the cost of such systems is still at such a high level that the popularity of mobile mapping systems remains limited, especially due to the price of the Inertial Measurement Unit (IMU). However, advances in MEMS technology have enabled the development of complete IMUs composed of multiple MEMS-based accelerometers and gyroscopes [16,17]. In addition to their compact and portable size, the price of MEMS-based systems is far less than those of high quality IMUs.

In the classical approach, the KF is applied in real-time applications to fuse different data from various sensors while optimal smoothing is applied in the post-mission mode. The basic idea of using the KF in INS/GPS integration is to fuse independent and redundant sources of navigation information with a reference navigation solution to obtain an optimal estimate of navigation states, such as position, velocity and orientation. However, the limitations of the KF have been reported by several researchers [12–15]. The major inadequacy related to the utilization of the KF for INS/GPS integration is the need for a predefined accurate stochastic model for each of the sensor errors [14]. Furthermore, prior information about the covariance values of both INS and GPS data as well as the statistical properties (*i.e.*, the variance and the correlation time) of each sensor system has to be accurately known [13–15]. Furthermore, for INS/GPS integration applications where the process and measurement models are nonlinear, the Extended KF (EKF) also work for nonlinear dynamic systems with a non-Gaussian distribution, except for heavily skewed nonlinear dynamic systems, where EKF may experience problems [18]. As indicated in [18], the EKF simply applies the first order term of the Taylor series expansion for the approximation of a nonlinear system and the probability density function is approximated by a Gaussian distribution [14,19]. Only small errors are allowed during estimation and the presence of nonlinear error behavior might violate the assumption thus generates biased solutions [19]. As indicated in [20], second order filters are able to compensate the bias term mentioned above but the computation burden of hessian (second order derivatives) is high.

**Figure 2.**
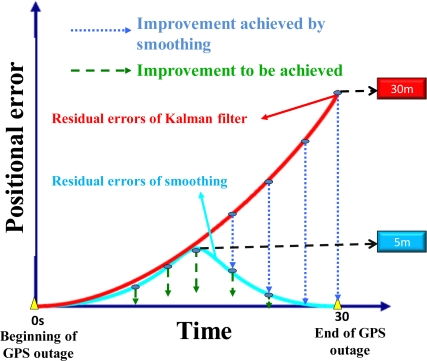
The impact of KF’s limiting factors on positional error during GPS signal blockage.

These limitations, in turn, may result in sub-optimal performance or even filter divergence if the assumption of local linearity is violated [18–20]. Each of these limiting factors contributes to a certain amount of positional error accumulation during GPS outages, as shown in Figure 2. In fact, the error behavior of orientation parameters during GPS outages is similar to the positional error shown in Figure 2 [11–13]. The scale of the maximum positional drift shown in Figure 2 is given based on the average value of the MEMS IMU applied in this study. Unfortunately, in modern urban canyons GPS signal blockages occur frequently. The magnitudes of the positional and orientation errors depend on the quality of the inertial sensors, the length of GPS outage, the dynamics of vehicle and the effectiveness of the algorithms applied. In other words, proper modification of inertial sensors or sensor fusion algorithms can reduce the magnitude of accumulated positional and orientation error during frequent GPS outages. Therefore, the goal of developing an alternative INS/GPS integration scheme is to reduce the impact of remaining limiting factors of KF and improve the positioning accuracy during GPS outages, which is critical for land-based mobile mapping applications.

In addition to these limitations, the problem of poor observation of inertial error states becomes the most critical issue, especially when integrating a low cost MEMS IMU with GPS [20,21]. Poor observation prevents the separation of the linear errors induced by accelerometers from angular errors induced by the gyroscopes and alignment errors due to the lack of motion dynamics [20]. This is a typical problem for low-cost IMUs as the motion dynamics are generally insufficient to separate linear and angular error terms during the correlation time of low-cost sensors [20].

Optimal smoothing algorithms, also known as smoothers, have been applied for the purpose of accurate positioning and orientation parameter determination through post-processing for most of surveying and mobile mapping applications with integrated sensors [13,14]. In contrast to the KF, the smoothing is implemented after all KF estimates have been solved by the use of past, present and future data. As shown in Figure 2, the magnitudes of positional and orientation errors during GPS outage can be improved significantly after applying one of these optimal smoothing algorithms. However, the magnitude of residual error shown with blue line also depends on the quality of the inertial sensors, the dynamics of vehicle and the length of GPS signal outage [13,22]. Therefore, the reduction of remaining positional and orientation errors becomes critical when integrating a low cost MEMS IMU with GPS for land based mobile mapping applications.

## 2. Problem Statements

As mentioned in the previous section, the accuracies of the KF solutions sometimes cannot fulfill the requirements of mobile mapping applications. An integrated system has to predict state parameters such as position, velocity and orientation using KF when GPS signal blockages appear [13]. But in such situations, the navigation solutions errors increase rapidly until the GPS signal is recovered to provide a measurement update, as shown in Figure 2. This problem becomes more critical when a low cost MEMS IMU is used. However, due to their noisy measurements and poor stability, the performance of current MEMS IMUs does not meet the accuracy requirements of mobile mapping applications [22].

In order to achieve high accuracy for positioning and orientation determination in mobile mapping applications, the data is processed in post-mission mode with an optimal smoothing algorithm. Most of the commercial mobile mapping systems use an optimal smoothing algorithm to provide accurate information on position and orientation for direct geo-referencing [10,22]. However, commercial INS/GPS integrated systems use tactical grade IMUs or above to provide accurate solutions for general mobile mapping applications. Therefore, upgrading the hardware (e.g., IMU) can be considered as an effective solution to improve the accuracies of position and orientation parameters when a low cost MEMS IMU is used. Still, this improvement is rather limited, as the availability of high grade (navigation) IMU is regulated by the government regulations of certain countries where those units are produced.

**Figure 3.**
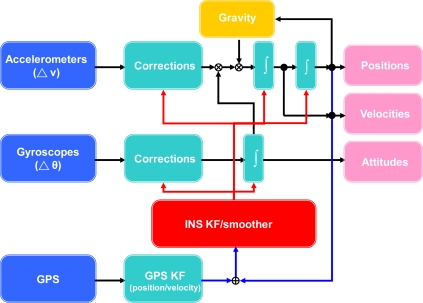
A loosely coupled INS/GPS integration architecture (closed loop).

Another effective way to improve the accuracies of low cost MEMS INS/GPS integrated solutions is through the improvement of sensor fusion algorithms. Compared to the hardware perspective mentioned above, the software perspective can be considered as a cost effective solution to develop a low cost sensor fusion solution for certain mobile mapping applications. Figure 3 illustrates the loosely coupled INS/GPS integration scheme commonly applied by most of the commercial mobile mapping systems [12,13].

The process of the KF is divided into two groups, those for prediction and updating [12,13]. The time prediction equations are responsible for the forward time transition of the current epoch (k-1) states to the next epoch (k) states. The measurement update equations utilize new measurements into the prior state estimation to obtain a state estimation *a posteriori*. The updated KF engine is triggered at every GPS measurement using the difference between GPS and INS solutions as input. Hence, the KF generates an updated estimate for reducing the INS errors using measurement update equations. Whenever GPS measurements are not available, the KF works in the time prediction mode to estimate the error state vector. The optimal smoothing is performed after the filtering stage and thus it relies on the previously filtered solutions. Consequently, an accurate filtering procedure is required for accurate smoothing process [13,14]. A fixed-interval smoother, the Rauch-Tung-Striebel backward smoother is implemented in this study. In fixed-interval smoothing, the initial and final time epochs of the whole period of measurements (*i.e.*, 0 and N) are fixed. Compared to other fixed-interval smoothers, the Rauch-Tung-Striebel backward smoother has the advantage of being the simplest to implement [13,14]. It consists of a forward sweep and a backward sweep. The forward sweep is the common KF with all predicted and updated estimates and corresponding covariances saved at each epoch of the whole mission. The backward sweep begins at the end of the forward filter (*i.e.*, at epoch N), see [13,14] for details.

The smoothed estimates at any epoch k are computed as a linear combination of the filtered estimate at that epoch and the smoothed estimate at the heading epoch k + 1. Thus, these smoothed estimates can be considered as updating the forward filtered solution to obtain improved estimates. The computation of the smoothed estimates at each epoch requires the storage of the KF predicted and updated (filtered) estimates and their corresponding covariances at each epoch [13,14]. This is the case in INS/GPS integrated solutions when uninterrupted GPS data streams are available. During GPS outages, only predicted estimates and covariances are available, a post-mission smoother can significantly remove the residual errors of KF [10,22]; however, some residual errors still remain, as shown in Figure 2. Therefore, the error behavior shown in Figure 2 has motivated various studies concerning the development of alternative multi-sensor fusion algorithms to reduce the magnitude of accumulated positional and orientation errors during frequent GPS outages in land applications.

## 3. Recent Development of Alternative Multi-Sensor Integration Algorithms

Three approaches concerning the development of alternative multi-sensor integration algorithms to improve the ability of analysis and prediction of complicated kinematic and nonlinear models to reduce the magnitude of accumulated positional and orientation errors during frequent GPS outages in land applications have been identified [22]. The first approach is known as sampling filter approach, such as particle filters [18,23–25] and unscented Kalman filter [22] while the second approach is known as artificial intelligence approach, such as the use of ANNs [26–28] or adaptive neural fuzzy information systems [29]. In addition, the third approach is known as the hybrid approach that combines conventional EKF or smoother based approaches with artificial intelligence approach [30,31].

The advantage of the sampling based approach is that the computation of derivatives is not applied [22]. Particle filters, also known as Sequential Monte Carlo filters, have been developed for nonlinear/non-Gaussian processes based on Bayesian filtering theory [32,33]. Gordon *et al*. [18] indicated that particle filters have a number of advantages that make them attractive for navigation applications; they are non-parametric, they can cope with nonlinearities and non-Gaussian noises, and are relatively easy to implement. Furthermore, the particle filter implementation does not require any assumption about the form of the posterior distributions [24]. Particle filters give an approximate solution to an exact model, rather than the optimal solution to an approximate model which is the basic for KF [34].

Bergman [23] applied a particle filter for INS/GPS integrated terrain navigation application and provided superior estimation accuracy than other methods applied because it does not make any assumption on the probability density function. Van Der Merwe *et al*. [35] proposed extended/unscented particle filters to incorporate the latest observation into a basic particle filter. The extended particle filter increases the processing time 2–5 fold, as compared to the EKF [36]. Moreover the performance of extended particle filter is highly dependent on the placement of the GPS outages. The extended particle filter performed 5–19% better during GPS outages in high dynamics areas, when compared with low dynamics GPS outages [36].

Aggarwal [24] proposed a hybrid extended particle filter as an estimation technique for integrating GPS and low cost inertial sensors. The performances are compared to those of the commonly used EKF using INS/GPS land-vehicle data set collected for low cost MEMS IMUs. The results show that both hybrid extended particle filter and EKF provide comparable navigation results during periods without GPS outages. However in cases when 60 second GPS outages are simulated, the hybrid extended particle filter performed much better than the EKF, especially when simulated outages lie in high vehicle dynamic areas; see [24] for a detailed numerical comparison.

According to Kubo and Wang [25], the Gaussian sum filter is also considered as a candidate for INS/GPS integration applications. The Gaussian sum filter is a nonlinear filter where its predictive *a priori* density is assumed to be the sum of several normal distributions. However the first order Taylor series approximation is applied for updating each distribution similarly to the EKF. Therefore, it suffers the impact of high nonlinearity similarly to the classic EKF. Kubu and Wang [25] proposed a strategy to combine the Gaussian sum filter and particle filter. It was developed based on the similar concept of the Gaussian sum filter, but updates its Gaussian sum expressions by using the particles instead of the linear approximations. The performance was compared with other filters in numerical simulations. From the simulation results, it is found that the Gaussian sum particle filter has the ability to improve the navigation performance when the initial estimates are provided with large uncertainty, see [25] for detailed numerical comparison of Gaussian sum particle filter and other filters.

To overcome the limitations of EKF instabilities and Jacobian evaluations, Julier and Uhlmann [37] proposed the unscented KF which deterministically generates a fixed number of minimal points, known as sigma points, from the Gaussian distribution, which estimate the true mean and covariance of Gaussian distribution. These sigma points are then individually propagated through the nonlinear system, to capture posterior mean and covariance accurately. Generally speaking, it is based on the hypothesis that it is easier to approximate a Gaussian distribution than to approximate an arbitrary nonlinear function [37]. Van der Merwe *et al.* [35] applied the unscented KF to improve the performance of the particle filter for an INS/GPS integrated system for unmanned aerial vehicle applications. As indicated in [22], the distinction between the unscented KF and particle filters is that the former takes samples deterministically while the later select samples randomly. Shin [22] also compared numerical performances between EKF/smoother and unscented KF/smoother with the use of various INS/GPS integrated systems in land applications.

**Figure 4.**
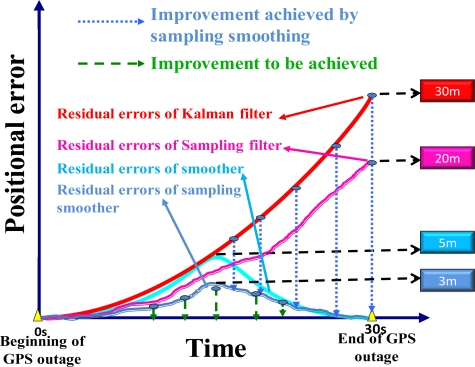
The error behaviors of sampling filters and smoothers during GPS outages.

Generally speaking, the results shown in [22–25] illustrate that the sampling filter approach and EKF based approach provide comparable navigation results during periods without GPS outages. However in cases when GPS outages are simulated, sampling filter approaches perform much better than the EKF, especially when simulated GPS outages lie in high vehicle dynamic areas [22,24]. The error behavior summarized from various sampling filters presented in [22–25] is shown in Figure 4. The positional errors of sampling filters, shown in red solid line, and the smoothers implemented with these sampling filters, shown in green solid line, resemble those of EKF and smoother, shown in dark and light gray lines, but with a reduced scale. It means that these sampling filters and their smoothers can reduce the positional and orientation error during GPS outages compared to conventional EKF and smoother, see [22–25] for details about their numerical ratios of improvement. Shin [22] indicated that the unscented Kalman smoother is able to provide 20%∼30% improvement in terms of positional and orientation accuracies during GPS outages compared to the smoother implemented with EKF. The scale of the maximum positional drift shown in Figure 4 is given based on the average value of the MEMS IMU applied in this study [22–25].

The artificial intelligence approach distinguishes itself from other types of estimation approaches by using inexplicit models, known as black box, to approximate the nonlinear relationships between system dynamics and measurements [22]. Chiang *et al.* [26] proposed a multi-sensor integration approach for fusing data from an INS and Differential GPS (DGPS) utilizing MFNNs for land applications. In addition, it addressed the impact of NN parameters and random noise on positioning accuracy of the integrated system especially during GPS outages. The experimental results demonstrate the advantages of new approach in terms of performance and computational efficiency. The field tests clearly show that once the architecture proposed in [26] is trained for about 1,800 seconds and becomes stable, position errors of less than 3 meters can be achieved, even beyond a GPS signal blockage of 200 seconds (*i.e.*, IMU stand-alone mode). These results are well beyond the performance characteristics expected from the IMU used in the NovAtel BDS GPS/IMU system.

Chiang *et al.* [38] introduced a window-based weight updating method and its use in ANNs for integrating GPS and INS in vehicular navigation applications. It was developed utilizing the stored weights and GPS signal window concept to overcome the limitations of traditional weight updating methods. Combing the latest GPS window signals, the stored weights can be adaptively updated to follow the latest motion dynamics and INS error characteristics. Hence it improves prediction accuracy during GPS outages. In addition to the significant improvement in positioning accuracy, the results demonstrate that the training time was far less than a GPS window length with the utilization of stored weights. Generally speaking, the INS/GPS ANN-based integration architectures proposed in [38] have provided positioning accuracies far better than those obtained through the use of conventional Kalman filtering integration.

Wang *et al.* [27] presented an ANNs-aided adaptive Kalman filter based on covariance matching technique for integrated INS/GPS systems. Instead of using a limited window for estimation as conventional adaptive KF, all the previous samples are counted in according to their character using ANNs. The covariance matching is conducted then its relation with the corresponding character is mapped with the ANNs. The adjustment of the adaptive KF is based on both the ANN training result and the updated covariance matching result. The test results show that the ANNs aided adaptive KF method can improve the navigation solutions. The ANNs applied after training can make reasonable predictions and is useful to improve the adaptive KF predictions.

Sharaf and Noureldin [28] introduced a new method for real-time INS and GPS integration in vehicular navigation utilizing radial basis function neural networks. This architecture is based on predicting the INS position error and continuously removing it from its corresponding INS position. This technique was employed in real time using special windowing technique. Results show the ability of their module to reduce the INS position error and prevent its growth even in the long term. In addition, it is able to accurately predict the INS position errors during GPS outages.

El-Sheimy *et al.* [29] developed a new design model for navigation applications using adaptive neuro-fuzzy inference systems to bridge periods of GPS signal blockage. This model uses neuro-fuzzy networks and the input/output patterns to train the fuzzy network during the availability of GPS solutions (which are used as a reference trajectory). During GPS signal blockage, the trained fuzzy model is implemented to predict the error drift of the standalone MEMS-INS estimated position. The performance of suggested model was compared to that of the traditional KF particularly during a number of simulated GPS outages. The test results indicate that the proposed neuro-fuzzy model can efficiently predict the INS errors during GPS outages.

Although artificial intelligence approaches are easier to design and implement, there are also limitations to these types of approaches [22]. Most of the approaches mentioned do not apply any statistical information during training process and they do not provide the statistics associated with the solutions produced by them. In other words, they lack the ability to provide proper accuracy measures concerning the navigation solutions provided by these artificial intelligent models. An ANN with the optimal topology is expected to provide the best approximation accuracy to the unknown model using the most appropriate number of hidden neurons and hidden layers. The empirical training for guessing the most appropriate topology is time consuming.

In addition, if the dynamics experienced by the vehicle exceeds the ranges of training data significantly, the performance of these approaches tends to deteriorate accordingly. Therefore, a frequent re-training procedure is required to guarantee the performance of these approaches. Chiang *et al.* [38] proposed the idea of navigation database and proper training strategy for the training procedure. Similar procedure was implemented in [28] for real time processing, including online weight updating. However, the accumulation and evolution of navigation knowledge require large amount of training data and they are time consuming [38]. In addition, these models require relatively large storage space compared to other estimation approaches.

**Figure 5.**
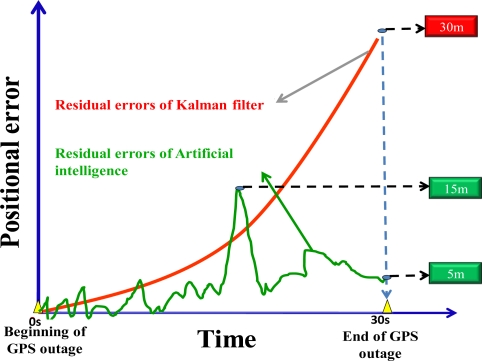
The error behavior of artificial intelligent models during GPS outages.

The navigation parameters provided by these artificial intelligent models are limited to positional parameters only because they rely on GPS solutions to provide training target and the use of a GPS receiver is unable to provide any reliable orientation parameters. Therefore, all of these artificial intelligent models are implemented to provide 2-D or 3-D positional parameters and bridge the gaps during GPS outages for land vehicular navigation applications [26–29,38]. However, it is not enough for mobile mapping applications because they require positional and orientation parameters simultaneously. Figure 5 illustrate the error behaviors of artificial intelligent models during GPS outages summarized from [26–29,38]. For short period of GPS outage, such as less than 10 seconds with the use of a MEMS IMU, the EKF is able to outperform artificial intelligent models. However, as the period of GPS outage extends to more than 30 seconds, artificial intelligent models outperform the EKF significantly [26–29]. On the other hand, unexpected vehicle dynamics result in the rapid growth of positional and orientation errors, as shown in Figure 5. The scale of the maximum positional drift shown in Figure 5 is given based on the average value of the MEMS IMU applied in this study [26–29].

**Figure 6.**
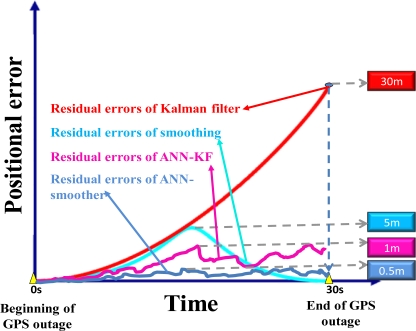
The error behaviors of hybrid model during GPS outages.

The hybrid approach is implemented by combing conventional EKF or smoother based approaches with artificial intelligent models. These artificial intelligent models are applied to model the error behaviors of conventional EKF or smoother and compensate for the errors of the positional and orientation parameters estimated by KF and Smoother during GPS outages. Goodall *et al.* [30] proposed a hybrid architecture that consists of ANNs and EKF to predict the error functions in a more optimal manner than one of the individual approaches. It is able to learn from its past errors and adapt to its application using radial basis functions networks, but retains the short-term estimation accuracy with aid of an EKF. Such an integrated approach has the capability of estimating all navigation states, using the advantages of ANN techniques for practical solutions. However, the accuracy requirements of general mobile mapping applications can’t be achieved easily even by the use of ANN-KF scheme. Therefore, Chiang *et al.* [31] proposed an intelligent position and orientation determination scheme that embeds MFNN with conventional smoother to improve the overall accuracy of a MEMS INS/GPS integrated system in post-mission mode. For the low cost MEMS system with the proposed ANN-smoother compensation, the positional and orientation parameters estimated by smoother can be improved to the level of using a medium tactical grade inertial system. Figure 6 illustrates the error behaviors of hybrid models during GPS outages summarized from [30,31]. The use of MFNN -KF schemes is able to improve the positioning and orientation accuracies to the level of the conventional smoother and the use of MFNN-smoother scheme is able to improve the positioning and orientation accuracies by 50% in average compared to the conventional smoother based on the experiment data provided in [30,31]. The scale of the maximum positional drift shown in Figure 6 is given based on the average value of the MEMS IMU applied in this study.

Generally speaking, a MFNN with an optimal topology is expected to provide the best approximation accuracy to the unknown model using the most appropriate number of hidden neurons and hidden layers [39,40]. There are many ways to decide the most appropriate number of hidden neurons [39,40]. The common principle indicates that the most appropriate number of hidden neurons is application dependent and can only be decided empirically during early stages of the topology design. It is very common to train many different candidate networks that have different numbers of hidden neurons and then to select the best, in terms of the performance based on an independent validation set in the design phase of ANN methodology [40]. Once the optimal topology is decided, the only free parameters to be adjusted during the re-training phase with the latest acquired training data are the weight values, but the topology remains fixed [41]. However, alternative schemes are expected to reflect the impact of new information to catch the latest dynamics and sensor error variation based on the characteristics of the INS/GPS integration applications. In an ANN terminology, this can be implemented by using a continuous learning process to adjust the weights with appropriate variation of topology to accumulate knowledge, if applicable [41,42]. Therefore, empirical guessing and fixed topology can be considered as the primary limiting factors of applying MFNNs for INS/GPS integration applications.

To avoid these limitations, several methods have been proposed in the last two decades to construct a neural network successively and automatically during the learning process. These methods are often recognized as constructive networks. The common principle is to start from a small network and then add hidden neurons and hidden layers as needed during the learning procedure using special algorithms. In other words, the networks are able to decide the appropriate topology based on the task given without human intervention. An overview of current constructive algorithms can be found in [41]. Among these algorithms, the CCNN algorithm proposed by Fahlman and Lebiere [42] has attracted the most attention.

Consequently, the objectives of this article were to: (1) develop a CCNN-smoother scheme for precise sensor positioning and orientation, (2) verify the performance of the proposed scheme using a low cost MEMS INS/GPS integrated system and (3) compare the performance with previously developed MFNN-smoother scheme in terms of the topology applied and estimated accuracy during GPS outages.

## 4. Artificial Neural Networks

**Figure 7.**
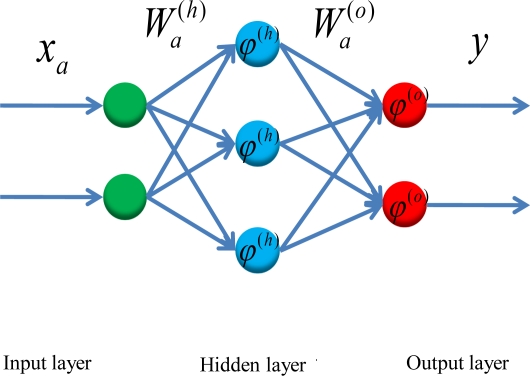
An example of a MFNN.

ANN methodologies have been motivated by the recognition that the human brain works in an entirely different way from a conventional digital computer [39]. The simplest form of an ANN can be depicted like the human nervous system. The receptors are used to convert input signals into appropriate vector that can be processed by a central network, while the effectors are used to transfer the output vector into readable response [40]. In general, the basic model of a neuron contains three major components: (a) weight links, (b) an adder for summing the input signals that are weighted by the respective synapses of the neuron, and (c) an activation function *ϕ*(•) for limiting the amplitude of the neuron output and the final output [39,40]. Figure 7 illustrates an example of a MFNN composed of one hidden layer and output layer, respectively. The input vector is expressed as *x_a_* and the weight links connecting input and hidden layers are given as a weight matrix, $W_{a}^{\left(h\right)}$. Similarly, the weight links connecting hidden and output layers are given as a weight matrix, $W_{a}^{\left(o\right)}$. The activation functions applied by hidden and output neurons are labeled as *ϕ*^(^*^h^*^)^ and *ϕ*^(o)^, respectively.

Therefore, the outputs of hidden and output layers, *Z_a_* and y, are given below:

(1) $$\mathrm{hidden}\{\begin{array}{c} s^{\left(h\right)}=W_{a}^{\left(h\right)}x_{a}\\ z_{a}=\varphi^{\left(h\right)}(s^{\left(h\right)}) \end{array}$$

(2) $$\mathrm{output}\{\begin{array}{c} s^{\left(o\right)}=W_{a}^{\left(o\right)}z_{a}\\ y=\varphi (s^{\left(o\right)}) \end{array}$$

where *s^(h)^* and *s^(o)^* represent the outputs of the adders in hidden and output neurons, respectively.

**Figure 8.**
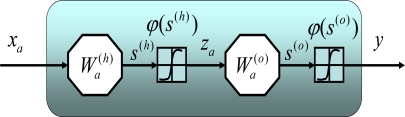
The nonlinear mapping capability of a MFNN.

Figure 8 illustrates the nonlinear mapping relationship between input and output vectors represented by the MFNN shown in Figure 7. To determine the optimal weight values ( $W_{a}^{\left(h\right)}$, $W_{a}^{\left(o\right)}$) one must have a set of examples of how the outputs, *y* should relate to input vector, *x_a_*. The process of obtaining these weights from these desired examples is called supervised learning and it is basically a conventional estimation or approximation process [40]. That is, these weight values are estimated from existing examples in such a way that the network, according to some metric, approximates the true relationship as accurate as possible [39,40].

**Figure 9.**
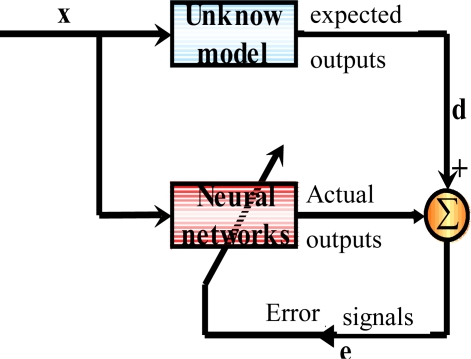
Supervised learning for function approximation.

Figure 9 illustrates the concept of supervised training strategy whereby the network develops based on inputs and observed outputs. It’s like a teacher who has knowledge of the environment, with that knowledge being represented by a set of input-output examples. Then the teacher and the neural network are both exposed to a training vector drawn from the environments, the teacher is able to provide the neural network with a desired response. The difference between desired response and neural network’s output will generate error signal and fed back to the neural network. The network parameters (weight and bias) are adjusted under the combined influence of the training vector and the error signal iteratively step-by-step to minimize the difference between desired response and neural network’s output [40].

A simplest way to understand how to adjust weight is to use standard backpropagation learning algorithm whose error function E is given below [40]:

(3) $$E=\frac{1}{2}{\left(t-y\right)}^{2}=\frac{1}{2}\sum_{k=0}^{j}{\left(d_{k}-y_{k}\right)}^{2}$$

Then the weight update formation is given as [40]:

(4) $$\Delta w=-\eta \frac{\partial E(w)}{\partial w}$$

where *η* is learning rate parameter

The primary goal of developing ANN-aided schemes is to improve the positioning and orientation accuracies during frequent GPS outages, which are crucial for land mobile mapping applications. As shown in Figure 2, the general error behaviors of the EKF grows systematically with time and that of smoother grows systematically first then decreases after reaching the peak, which usually takes place in the middle of GPS outage. These behaviors are coupled with vehicle dynamics, inertial sensor errors, the length of GPS outage and the quality of the system and measurement model applied. However, these error behaviors are too complicated to describe through proper mathematical models [28]. The general idea of ANNs is to build up the nonlinear mapping relationship between inputs and outputs and learns for examples and generalizes for applications.

Therefore, the inputs applied in this study include positional and orientation states estimated by the EKF and smoother along with time information while the outputs include the errors of those estimated states during GPS outages. In other words, the proposed topology realizes the nonlinear mapping relationships between system dynamics, the length of GPS outage and the error behaviors of the EKF and smoother during GPS outages. Through using proper training strategy with sufficient training data, the proposed scheme is able to generalize the nonlinear mapping relationships between system dynamics, the length of GPS outage and the error behaviors of the EKF and smoother during outages.

In this study, one of the constructive ANNs, the CCNN, is implemented to learn and compensate for the residual errors of the KF and smoother, respectively, to improve the accuracies of the positional and orientation parameters. The proposed scheme is capable of learning how the state vector (*i.e.*, position or orientation errors) behaves under the influences of dynamics of the platform, the error characteristics of the inertial sensors being used and the length of GPS outage applied. As mentioned previously, the residual error compensation scheme developed involves a set of complicated non-linear function approximations to adapt to the influence of the variations of vehicle dynamics and sensor errors on KF and smoother during GPS outages [26,28].

Two key ideas in implementing the CCNN algorithm include a cascade architecture and unique learning algorithm for automatically training and installing new hidden neuron [42]. The optimal values of weight are computed during the training process.

**Figure 10.**
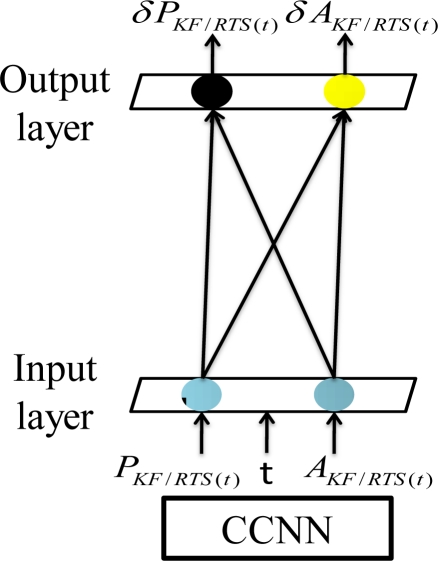
The initialization of a CCNN.

The CCNN architecture starts with a minimal topology, consisting of only the input and output neurons. The optimal values of the direct input-output weight links are computed during the training procedure, and the training continues with a minimal topology for the entire training data set until no further improvement can be achieved, as shown in Figure 10. During this process, there is no need to back propagate the output error through hidden neurons. Any conventional training algorithm for a single layer feed forward neural network (e.g., the standard gradient descent algorithm) can be applied.

**Figure 11.**
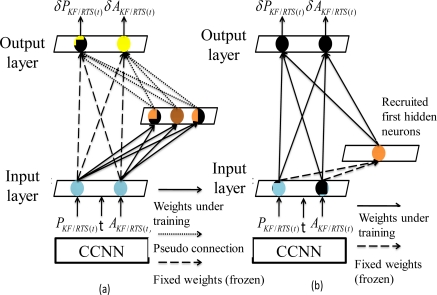
The recruitment and installation of the first hidden neuron.

To recruit a new hidden neuron, a pool of candidate neurons that have different sets of randomly initialized weight values is applied. All these candidate neurons within the pool receive the trainable input connections from the external inputs. In addition, they receive the same residual error for each training pattern sent from the output neurons through the pseudo connections shown in Figure 11a. Thus, the recruitment of the first hidden neuron can be completed in a two-step process.

During the first step of recruitment, each candidate neuron is connected to each of these input neurons, but is not connected to the output neurons. The primary task of pseudo connections shown in Figure 11a is to deliver residual error for each training pattern to these candidate hidden neurons. The weight links connecting the input and candidate neurons are adjusted to maximize the correlation between the output of each candidate neuron and the residual error at the output neuron, as shown in Figure 11a. Meanwhile, the weight links connecting input and output neurons remain fixed. A number of passes over the training data are executed and the weights connecting to the inputs of these candidate neurons are adjusted after each pass. The weights between the candidate layers, input layer are adjusted to maximize the correlation between the output of each candidate neuron and the residual error at the output neuron [42]. The Quickprop algorithm is applied to adjust incoming weights until there is no more improvement in each candidate neuron’s correlation [42]. The candidate neuron with the highest correlation is recruited in the topology as a new hidden layer, as shown in Figure 11b.

The second step of recruitment process initializes after the neuron with the highest correlation is installed in the topology as a new hidden layer. Only one layer of weights is trained during the second step of recruitment process shown in Figure 11b. The incoming weights of the winning candidate neuron are frozen and it is recruited and then inserted into the active network in the second step of recruitment. The newly added hidden neuron is then connected to the output neurons and the weights connection become adjustable. All connections to the output neurons are trained, as shown in Figure 11b. In other words, the frozen weight links that connect the input and output neurons shown in Figure 11a are trained again using the Quickprop algorithm [42], as illustrated in Figure 11b. In contrast, the weight links connecting the recruited first hidden neuron and output neurons are trained for the first time. The recruitment and installation of the second hidden neuron initializes when the training goal cannot be met with current topology composed one hidden neuron.

**Figure 12.**
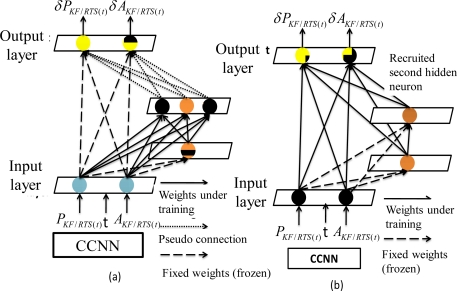
The recruitment and installation of the second hidden neuron.

The second hidden neuron is then recruited using the process shown in Figure 12. Similarly, each candidate neuron from the pool is connected to each of these input neurons, but is not connected to the output neurons during the first step of recruitment. The weights connecting the input neurons, pre-recruited hidden neurons and candidate neurons are adjusted to maximize the correlation between the output of each candidate neuron and the residual error at the output neuron, as shown in Figure 12a. These candidate neurons receive input signals from both input neurons and the previously recruited hidden neuron. All weights connecting the inputs, pre-requited and candidate hidden neurons are adjusted to recruit the second hidden neuron. The neuron with the highest correlation is recruited in the topology as the second hidden layer, as shown in Figure 12b. The values of these weights connecting to these inputs and pre-recruited hidden neurons are then frozen as soon as the second hidden neuron is added to the active networks. All these connections to the output neurons are then established and trained. As shown in Figure 12b, all of these pre-recruited hidden neurons can be regarded as additional input neurons during the training of the single layer of weight values.

**Figure 13.**
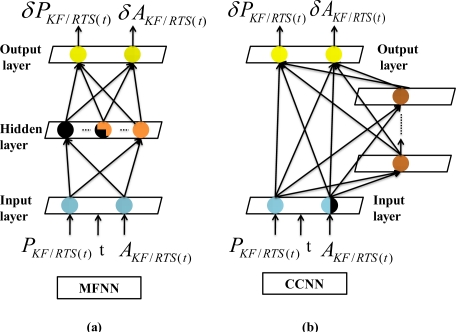
The comparison between finalized CCNN and MFNN topologies.

The process of recruiting new neurons, training their weights and training all connections to the output neurons, is continued until the errors reach the training goal or the maximum number of iterations or epochs (as defined by the user). According to [39], an epoch is defined when the presentation of the entire training set to the neural network (or hidden units) is reached. The finalized CCNN topology shown in Figure 13b becomes a modified version of original MFNN topology shown in Figure 13a with *n* hidden neurons and *n* hidden layers. In other words, each hidden layer consists of only one hidden neuron [42].

According to [43], the use of CCNNs for developing an alternative INS/GPS integration scheme has several advantages over MFNNs. First, the best topology can be decided automatically based on the complexity of the applications and there is no need to perform extensive empirical trials to determine the size and depth of the network (i.e., the number of hidden neurons and hidden layers, respectively). Moreover, the learning speed of CCNN is fast [42]. As indicated in Figures 11a and 12a, since only one layer of weight values are trained due to the fact that the recruited hidden neurons are treated as additional input neurons, the residual error signal can be delivered to all hidden neurons at the same time [42]. In addition, CCNN methodologies are useful for incremental learning, in which new information is added to a network that has been previously trained [41,42]. They can thus reflect variation in the model complexity by adjusting their weight values and topology automatically with additional information. In contrast, MFNNs can only alter the weight values to track the variations in model complexity [39,40].

**Figure 14.**
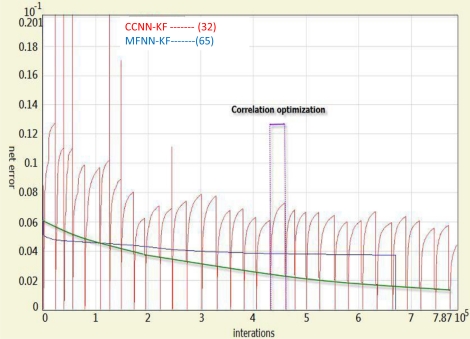
The comparison of the learning behaviors of CCNN-KF and MFNN-KF based schemes.

Based on the training data applied in this study, the topologies of the proposed schemes are shown in Table 1. It can be seen that proposed CCNN KF/smoother schemes use fewer hidden neurons than MFNN KF/smoother schemes. In addition, the inputs and outputs of MFNN based scheme are exactly the same as those of CCNN based schemes. However, the number of hidden neurons and layers of the MFNN based schemes are decided empirically. In contrast, the topologies of the CCNN based schemes grow automatically online. All these hidden neurons applied by both schemes are nonlinear (e.g., a hyperbolic tangent sigmoid) and all these output neurons applied by them are linear. Figure 14 illustrates the comparison of the learning behaviors of CCNN-KF and MFNN-KF based schemes based on the training data applied in this study. The red line represents the error produced by the CCNN-KF scheme during the first step of the procedure (correlation optimization) and the green line represents the error produced by the CCNN-KF scheme after completing the recruitment of a hidden neuron. Therefore, thirty-two learning patterns can be observed, as the procedure is repeated automatically for thirty-two times. In contrast, the blue line represents the error of MFNN-KF scheme. As shown in Figure 14, when a new neuron is inserted into the CCNN, its residual error reduces effectively.

As indicated in Figure 14, the CCNN-KF scheme converges faster than the MFNN-KF scheme with the same training data set and training goal. Table 1 also indicates the training speed of CCNN based schemes is faster than MFNN based scheme by 100% in average. Each hidden layer of the CCNN-KF scheme only consists of one hidden neuron, and thus their final topology becomes deeper than that of the MFNN-KF scheme (*i.e.*, they have more hidden layers). Based on the results presented in Figure 14, the CCNN-KF scheme is able to reach the same training goal with less training time and a simpler architecture compared to the MFNN-KF scheme. In addition, proposed CCNN based schemes are distinguished from MFNN based schemes as they can decide their latest topology “on the fly” based on the dynamic variations and inertial sensor errors if new training data is provided. As shown in Table 1, the learning time of MFNN based schemes is around 10 minutes when the numbers of hidden neurons equal to 60 and 65. However, during the empirical training process, the number of hidden neurons had been validated from 5 to 100 and this process took more than 3 hours to choose the appropriate numbers, which are 60 and 65, respectively. In fact, the increments of adding the number of hidden neurons to the new candidate neural networks was 5 thus the training processes were repeated for training 20 candidate neural networks. In other words, with identical training samples, the MFNN based schemes applied in this study are decided by the designer after extensive training process for at least 3 hours when the CCNN-based schemes applied in this study construct their topologies autonomously without human intervention within 6 to 8 minutes.

## 5. System Architecture

The EKF applied in this study has 21 states, which are given as follows:

$$[{\begin{array}{lllllll} \delta p_{1\times 3} & \delta v_{1\times 3} & \delta A_{1\times 3} & b_{a,1\times 3} & b_{g,1\times 3} & S_{a,1\times 3} & S_{g,1\times 3}] \end{array}}^{T}$$

**Figure 15.**
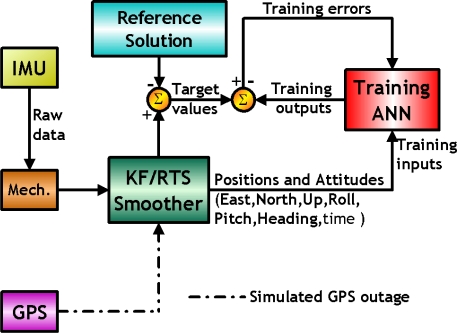
An ANN training architecture (adopted from [31]).

As shown in Figure 15, the EKF and smoother are utilized to optimally estimate these states and to compensate for their effects in real-time and the post-mission modes, respectively. In fact, either approach can provide optimally estimated nine navigation parameters. In addition, sensor biases (*b_a_*_,1×3_ and *b_g_*_,1×3_) and scale factors (*S_a_*_,1×3_ and *S_g_*_,1×3_) can be estimated and feed back to the INS mechanization to correct these raw measurements provided by an IMU. The scope of this study is to improve the accuracies of positional and orientation parameters during GPS outages, only the components concerning these parameters are shown in Figure 15. This means that the sensor errors are not included in the inputs to ANN schemes, as shown in Figure 15.

The errors of positional and orientation parameters estimated by the KF and smoother during GPS outages are used as the desired outputs or target values during the training process of various proposed ANN architectures, including MFNNs and CCNNs. The positional and orientation parameters estimated by the KF and smoother along with the time information in each scenario are used as the inputs of the proposed architectures. The goal of the proposed schemes is to compensate for the errors of the positional and orientation parameters estimated by the KF and smoother during GPS outages [30,31]. A superior IMU is applied as the reference system to generate reference solutions computed by the post-mission process (e.g., smoother) with the full availability of GPS. The target values are these errors of KF and smoother with intentionally added GPS outages compared to reference solutions [30,31].

An ANN with optimal topology is expected to provide the best approximation accuracy for the unknown model using the most appropriate number of hidden neurons and hidden layers [39,40]. The CCNN has a flexible topology, as mentioned before; so there is no need to design these two parameters through extensive training process. But with MFNN there are many ways to decide on the most appropriate number of hidden neurons [39,40]. The standard principle is to decide it empirically during the early stages of topology design. It is thus very common in the design phase of neural networks to train many different candidates’ networks that have different numbers of hidden neurons and then to select the best, in terms of its performance based on an independent validation set [39,40].

**Figure 16.**
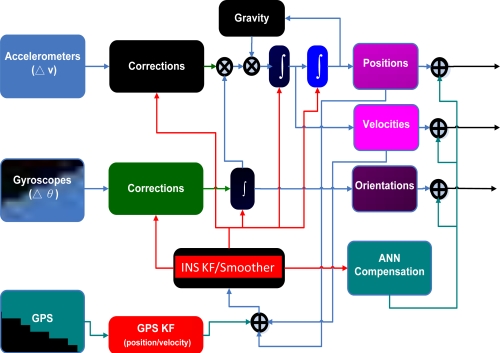
The implementation of ANN embedded KF and smoother (adopted from [31]).

After being well trained, the proposed ANN compensation schemes are added to a loosely coupled INS/GPS integration architecture (closed loop), as shown in Figure 16. The intelligent architecture first receives raw data from an IMU and then use the INS mechanization along 21 states of KF and smoother to estimate positional and orientation parameters, respectively. Meanwhile, the estimated positional and orientation parameters are sent to the proposed ANN architectures along with time information to generate predicted errors to compensate for the estimated positional and orientation parameters provided by the KF and smoother simultaneously. Errors in these positional and orientation parameters are predicted with the proposed ANN schemes, and the correction can be completed after the predicted errors have been removed from the outputs of the KF and smoother.

## 6. Results and Discussion

**Figure 17.**
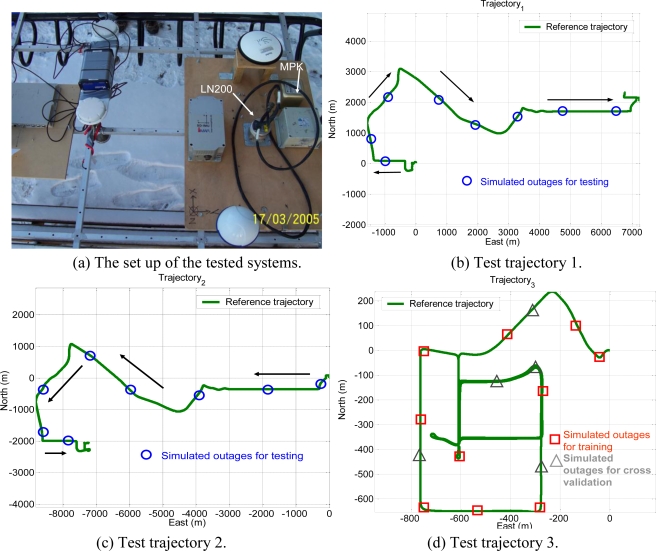
The tested systems and experimental trajectories.

Three field tests were used to evaluate the performance of the proposed schemes. The tests were conducted in land vehicle environments using different integrated systems consisting of one tactical grade IMU, Litton LN200 (1 deg/hr), a low cost MEMS IMU, BEI MotionPak II and two NovATel OEM-4 receivers. In this study, those IMUs were applied to collect inertial measurements in the field and then these along with carrier phase DGPS solutions were fed into software that has an inertial navigation algorithm and EKF to estimate inertial states optimally. The integrated system with LN200 IMU was used as the reference. The measurements and navigation solutions provided by the integrated system with MotionPak II were used to verify the performance of proposed schemes. Figure 17a shows the set up of these systems and illustrates the trajectories of the field tests. The experimental conditions are summarized in Table 2.

The GPS measurements were processed using GrafNavTM software (Waypoint Consulting Inc.) in carrier phase DGPS to achieve ten centimeter level accuracy. The reference trajectories were generated by the integrated system with LN 200 IMU. They were determined using 21-state EKF and smoother implemented in the Aided Inertial Navigation Software (AINS) from the Department of Geomatics Engineering at of the University of Calgary. These parameters of EKF and the smoother applied in this study were well tuned so that they can represent the best achievable navigation accuracy for tactical grade IMUs.

Ten GPS outages, marked with circles and each lasting 30 seconds, were simulated using the measurements collected in the third field test, as indicated in Figure 17d. The outputs of the KF and smoother provided by those systems were applied as the inputs for the proposed architectures. Several input dimensions were considered by choosing some of the outputs from the KF and smoother. In addition, the outputs of the KF and smoother with simulated GPS outages were then compared with the reference trajectory. The errors, which can be interpreted as the error behaviors of the KF and smoother, were then applied as the desired output for training. As shown in Figure 17d, the dynamics variations experienced by the vehicle during the simulated outages include straight line segments, sharp turns, accelerations and decelerations. It is worth noting that five simulated outages, marked with triangles, were used as the independent data set for cross validation during training process to ensure generalization capability as well as to avoid possible over-training problems.

In addition, a total sixty four GPS outages, each has 30 seconds in length, were simulated randomly in four scenarios using the measurements collected in the first and second field tests using the INS/GPS integrated with the MotionPak II (MEMS) IMU. Both field test data sets are applied to verify the performance of the proposed schemes.

### 6.1. The training of the proposed schemes

**Figure 18.**
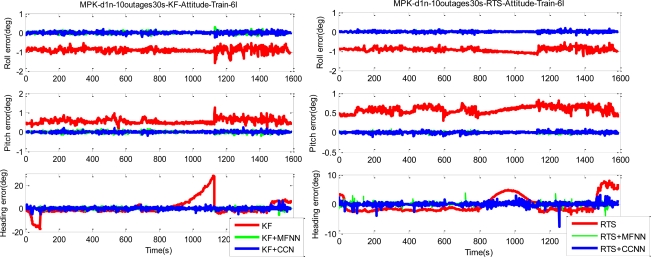

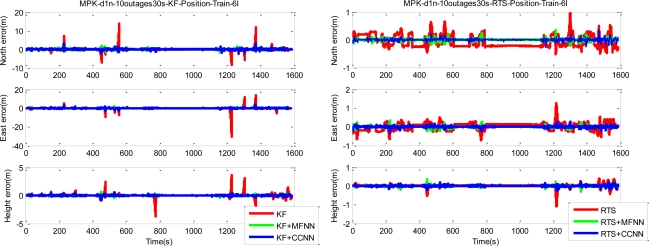
The samples of compensated positional and orientation errors (Training scenario).

Figure 18 illustrates the training results in terms of the errors of compensated positional and orientation solutions. Table 3, summarizes various statistical indexes including Root Mean Squared (RMS) errors, medians of errors and 99-Percentile of errors derived from Figure 18. As indicated in Figure 18 and Table 3, the proposed ANN-KF and ANN-smoother schemes learned the error behavior of the KF and smoother during simulated GPS outages well, especially the heading angles and height components. In addition, the proposed CCNN-KF/smoother schemes also learned the error behavior of the KF and smoother well and provided comparable performance to MFNN-KF/smoother schemes.

As show in Table 3, the RMS errors of CCNN-KF/smoother and MFNN-KF/smoother based schemes are reduced by 99% on average compared to KF/smother solutions. In addition, the median values of different errors of KF/smother solutions are reduced by 100% on average, which means that the proposed schemes remove the biases due to the setting issue between test and reference systems after training. In other words, the proposed schemes are able to compensate for the biases due to the setting issue between test and reference systems automatically as long as these systems are fixed on the same plate. In addition, the systematic error behaviors of positional and orientation parameters estimated by smoother during GPS outages, which are considered as the total impact of vehicle dynamics, inertial sensor errors and the length of GPS outrages, are fully compensated. Therefore, the median values of these errors produced by proposed schemes shown in Table 3 approach to zero.

Similarly, the 99-Percentile of errors of CCNN-KF/smoother and MFNN-KF/smoother based schemes are reduced by 99% on average compared to KF/smother solutions. However, the performance of the proposed schemes still needs to be verified using other independent data sets and this is presented in the next section.

### 6.2. Performance verification of proposed schemes

**Figure 19.**
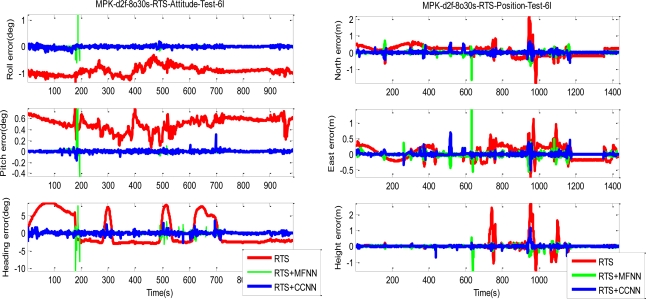
The samples of compensated positional and orientation errors (Testing scenario-I with trojectory_1).

**Figure 20.**
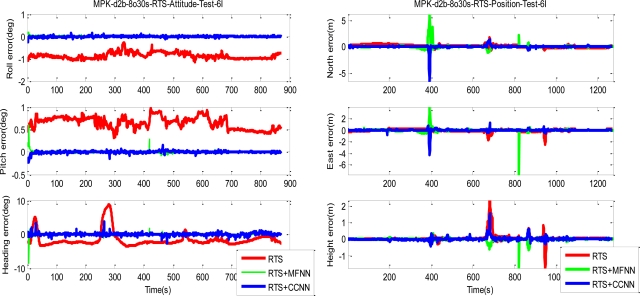
The samples of compensated positional and orientation errors (Testing scenario-I with trojectory_2).

**Figure 21.**
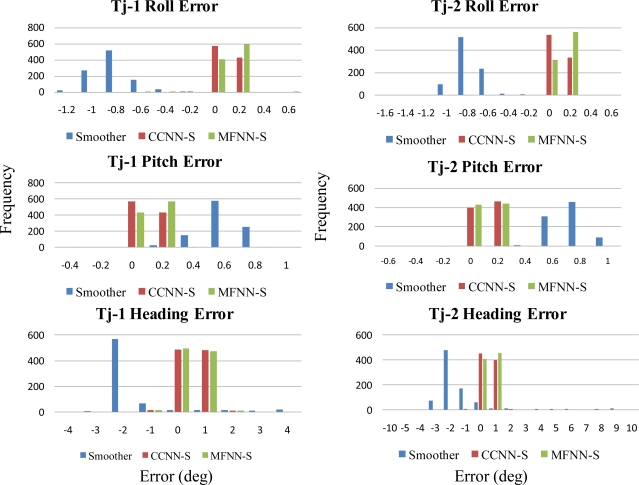
The histograms of orientation errors (Testing scenario-I).

**Figure 22.**
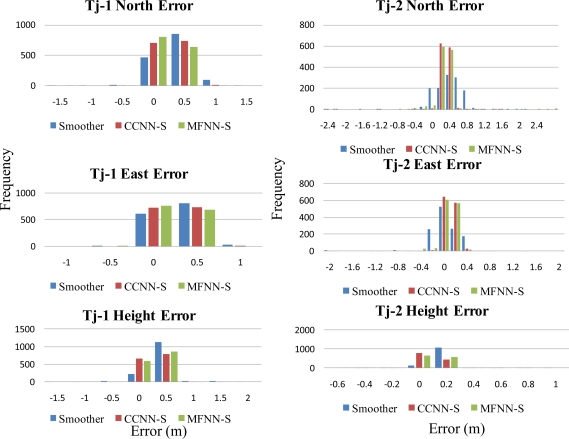
The histograms of positional errors (Testing scenario-I).

Since the objective of this study was to verify the performance of proposed CCNN-smoother and compare to previously developed MFNN-smoother and commercially available smoother based schemes, those results concerning CCNN-KF, MFNN-KF, and commercially available KF are not provided to condense the discussions of this study. To verify the performance of well-trained ANN-smoother schemes, four sets of testing scenario are created. Each scenario consists of eight simulated GPS outages (30 seconds each) which are randomly selected from test trajectory 1 and trajectory 2, respectively. Therefore, the proposed algorithms are validated with 16 randomly selected GPS outages in each scenario. Figure 17 illustrates those simulated GPS outages applied in the first testing scenario, which are marked in blue squares. Certain measures have been taken to avoid generating repeating GPS outage with the same time span in different scenarios. Therefore, the proposed algorithms are validated with four scenarios composed of 64 randomly selected GPS outages to examine the generalization of proposed algorithms. Since each field test was conducted independently, these simulated GPS outages selected from the first and second tests used for the verification were independent of those outages used for training. The dynamic variations experienced by the vehicle during the simulated outages include straight line segments, sharp turns, accelerations and decelerations. Figures 19 and 20 depict the comparison of compensated positional and orientation errors between proposed CCNN-smoother scheme, MFNN-smoother scheme and smoother using independent test data sets in with the first testing scenario. Figures 21 and 22 depict the histograms of orientation and positional errors produced by applied schemes with the first testing scenario.

As shown in Figure 19, the performance of MFNN-smoother deteriorates because of several undesirable peaks. These oscillations can be considered as the impact of overtraining or over-fitting during training stage, and they usually appear when the training goal is too small and thus the MFNN schemes learn the error behaviors of the INS/GPS integrated system based on the training data provided by trajectory 3 too well. In other words, the MFNN-smoother is trapped in the local minima which prevent their performance achieving better generalization using independent test trajectories. In addition, this issue is closely related to the fixed topology of MFNN-smoother applied.

Since it is decided through empirical trials during the training stage, the number of hidden layers and neurons are determined by the designer. Insufficient or excess hidden layers and hidden also lead to the impact of overtraining or over-fitting, although this can be mitigated by reducing the number of training epoch, increasing the number of training goal or adjusting the number of hidden neurons. However, the ratios of improvement are not as good as with the CCNN-smoother scheme. The number of training epochs for MFNN applied in this study is reduced from 500 to 200. As shown in Figures 19 and 20, the performance of the CCNN-smoother scheme is comparable to MFNN-smoother scheme. In addition, the results of the former are smoother than those of the latter because it avoids the impact of overtraining as it grows its topology automatically.

As shown in Figures 21 and 22, the histograms of positional and orientation errors produced by smoother scheme illustrate the impact of biases due to the setting of test and reference systems and the systematic error behaviors of positional and orientation parameters estimated by smoother during GPS outages, which are considered as the total impact of vehicle dynamics, inertial sensor errors and the length of GPS outrages are fully compensated. In addition, the positional and orientation error deviations or variations produced by smoother scheme are wider than those produced by proposed CCNN-smoother and previously developed MFNN-smoother schemes, which means the proposed schemes increase the stability of the system.

Generally speaking, the majority of positional and orientation errors produced by proposed CCNN-smoother and previously developed MFNN-smoother schemes concentrate around 0, which means that they are able to compensate various systematic errors automatically and increase the stability. On the other hand, the distributions of positional and orientation errors produced by CCNN-smoother and MFNN-smoother schemes are comparable.

Table 4a to 4d illustrate statistical summary of the compensated positional and orientation solutions from four testing scenarios applied in this study, respectively. Generally speaking, the proposed CCNN-smoother scheme output perform smoother applied in most of the commercial packages for mobile mapping applications in terms of RMS errors, medians and 99-Percentile of orientation errors by 80% in average. Similarly, it output performs smoother in terms of RMS errors, medians and 99-Percentile of positional errors by 60% in average.

On the other hand, the performances of proposed CCNN-smoother and previously developed MFNN-smoother schemes are similar in terms of RMS errors, medians and 99-Percentile of positional and orientation errors. The results presented in this study indicate that the proposed CCNN-smoother scheme is able to achieve comparable performance when undertaking the prediction task with fewer hidden neurons and less training efforts. In addition, based on the characteristics of the INS/GPS integration applications, the scheme applied is able to reflect the impact of new information and so catch the latest dynamic and sensor error variations. In a neural network, this can occur with a continuous learning process to adjust the weights and appropriate variation of topology if needed. Consequently, self-growing or construct networks such as CCNNs are better than fixed topology networks such as MFNNs.

The error behaviors of KF and smoother during GPS outage are coupled with vehicle dynamics, inertial sensor errors, the length of GPS outage and the quality of the system and measurement model applied. However, these error behaviors are too complicated to describe through proper mathematical models. The general idea of ANNs is to build up the nonlinear mapping relationship between inputs and outputs and learns for examples and generalizes for applications. Therefore, the proposed topology realizes the nonlinear mapping relationships between system dynamics, the length of GPS outage and the error behaviors of the EKF and smoother during GPS outages. Through using proper training strategy with sufficient training data, the proposed scheme is able to generalize the nonlinear mapping relationships between system dynamics, the length of GPS outage and the error behaviors of the EKF and smoother during outages

Therefore, the proposed CCNN-smoother scheme significantly improves the accuracies of all these positional and orientation parameters estimated by smoother applied in most of the commercial packages for mobile mapping applications for a low cost MEMS integrated system based on the field test data applied. Among these parameters compensated by proposed CCNN-smoother scheme, the improvement of the orientation parameters is more significant than that of positional ones. However, more training data sets are required in future studies to generalize the contributions of this study.

In addition, the use of CCNNs for developing an alternative INS/GPS fusion scheme has several advantages over MFNNs. First, the best topology can be decided automatically based on the complexity of the applications, and there is no need to perform extensive empirical trials to determine the size and depth of the network. Table 5 compares the strengths and weaknesses of MFNN and CCNN based schemes. Ultimately, the idea of using CCNN is mainly motivated by the limitations of MFNNs, which include extensive empirical trails, poor generalization, overtraining issue, fixed topology, poor adaptability to new training data, and poor knowledge accumulation mechanization. The preliminary results presented in this study illustrate CCNN based scheme reduce the impacts of those limiting factors mentioned above significantly. Therefore, it is considered superior in terms of the performance in ANN domain. In addition, it is able to provide comparable performance in application domain.

## 7. Conclusions

This study developed a CCNN embedded sensor fusion algorithm to improve the accuracy of positional and orientation parameters during GPS outages using a novel procedure that combines CCNN architecture and the smoother applied in most of the software packages for mobile mapping applications for post-mission processing.

The preliminary results presented in this study indicate that the proposed CCNN-smoother scheme outperforms commercially available smoother schemes using a low cost MEMS INS/GPS integrated system in terms of RMS errors, medians and 99-Percentile of orientation errors by 80% in average based on the field test data applied in this study. Similarly, it outperforms smoothers in terms of RMS errors, medians and 99-Percentile of positional errors by 60% in average. Among these parameters compensated by proposed CCNN-smoother scheme, the improvement of orientation parameters is more significant than that of positional ones.

On the other hand, the performances of proposed CCNN-smoother and previously developed MFNN-smoother schemes are comparable in terms of RMS errors, medians and 99-Percentile of positional and orientation errors. The results presented in this study indicate that the proposed CCNN-smoother scheme is able to achieve comparable performance when undertaking the prediction task with a simpler and flexible topology decided automatically and with less training efforts. However, more training data sets are required to generalize the contributions and validate the applicability of propose algorithms for real life applications in future studies.

## Figures and Tables

**Table 1.** The topology comparison of CCNN and MFNN based schemes.

**Topology**	**CCNN-KF**		**CCNN-smoother**		**MFNN-KF**		**MFNN-smoother**	
P: position. A: orientation	P	A	P	A	P	A	P	A
Input neurons	7	7	7	7	7	7	7	7
Hidden layer/neuron	32/32	35/35	34/34	35/35	1/60	1/65	1/60	1/65
Output neurons	6	6	6	6	6	6	6	6
Training time(s)	355	378	359	366	583	645	586	650

**Table 2.** The summary of the experimental conditions.

**Index**	**NVS**			**PDOP**			**Speed (m/s)**			**Date**	**Duration (seconds)**
	**Min.**	**Max.**	**Avg.**	**Min.**	**Max.**	**Avg.**	**Min.**	**Max.**	**Avg.**		
**Tj-1**	4	10	7	1.2	5.8	2.2	0	22	7.5	03.17.2005	2,400
**Tj-2**	4	10	7	1.1	5.8	2.1	0	22	8.2	03.17.2005	1,850
**Tj-3**	4	10	7.5	1.4	5.8	2.4	0	22	7.8	03.16.2005	1,700

NVS: Number of visible satellites, Min.: Minimum, Max.: Maximum, Avg.: Average, Speed: Horizontal velocity.

**Table 3.** Statistical Summary of the compensated positional and orientation solutions (Training scenario).

States		RMS			Median			99-Percentile		
		K	CCNN-K	MFNN-K	K	CCNN-K	MFNN-K	K	CCNN-K	MFNN-K
Tj-3	Roll(deg)	0.919	0.049	0.038	−0.932	0.002	0.003	0.611	0.039	0.048
	Pitch(deg)	0.573	0.04	0.034	0.623	0.003	0.004	0.521	0.028	0.034
	Heading(deg)	6.434	0.921	0.496	1.823	0.002	0.002	3.410	0.750	0.886
	North (m)	1.081	0.319	0.273	−0.250	−0.003	0.005	1.079	0.328	0.383
	East (m)	1.781	0.342	0.215	−0.364	0.004	−0.004	1.780	0.342	0.415
	Height (m)	0.365	0.077	0.068	0.636	0.006	−0.001	0.363	0.067	0.078
K: Kalman filter; CCNN-K: CCNN-Kalman filter; MFNN-K: MFNN-Kalman filter										
States		RMS			Median			99-Percentile		
		S	CCNN-S	MFNN-S	S	CCNN-S	MFNN-S	S	CCNN-S	MFNN-S
Tj-3	Roll(deg)	0.929	0.031	0.023	−0.932	−0.002	0.001	−0.246	0.028	0.050
	Pitch(deg)	0.586	0.020	0.019	0.595	0.001	0.003	0.302	0.046	0.041
	Heading(deg)	2.776	0.567	0.483	−0.345	−0.002	0.002	4.630	0.302	0.426
	North (m)	0.233	0.042	0.040	−0.08	0.002	0.003	0.757	0.292	0.348
	East (m)	0.217	0.039	0.047	0.045	0.003	−0.003	0.823	0.109	0.172
	Height (m)	0.103	0.036	0.034	0.021	0.002	−0.002	2.277	0.125	0.139
S: Smoother; CCNN-S: CCNN-Smoother; MFNN-S: MFNN-Smoother										

**Table 4.** Statistical summary of the compensated positional and orientation solutions.

**(a)**Testing scenario-I.										
States		RMS			Median			99-Percentile		
		S	CCNN-S	MFNN-S	S	CCNN-S	MFNN-S	S	CCNN-S	MFNN-S
Tj-1	Roll(deg)	0.935	0.043	0.052	−0.933	−0.003	0.002	−0.476	0.088	0.050
	Pitch(deg)	0.524	0.020	0.040	0.537	−0.002	0.001	0.702	0.046	0.041
	Heading(deg)	3.577	0.428	0.646	−2.198	−0.002	−0.003	8.630	1.302	1.426
	North (m)	0.359	0.104	0.108	0.187	0.001	−0.003	0.857	0.492	0.348
	East (m)	0.224	0.068	0.076	0.042	0.000	−0.001	0.623	0.309	0.172
	Height (m)	0.374	0.080	0.083	0.025	0.001	0.004	1.977	0.295	0.139
Tj-2	Roll (deg)	0.877	0.029	0.021	−0.894	−0.005	0.004	−0.442	0.059	0.069
	Pitch (deg)	0.664	0.026	0.030	0.678	0.001	0.000	0.901	0.045	0.095
	Heading(deg)	2.707	0.395	0.334	−2.266	−0.012	0.003	8.314	1.061	0.451
	North (m)	0.478	0.370	0.432	0.177	0.000	−0.002	0.651	0.536	1.853
	East (m)	0.361	0.257	0.325	−0.072	−0.001	−0.002	0.276	0.118	0.421
	Height (m)	0.261	0.101	0.090	0.030	−0.005	−0.002	1.393	0.449	0.086
S: Smoother; CCNN-S: CCNN-Smoother; MFNN-S: MFNN-Smoother										

**(b)** Testing scenario-II.										
States		RMS			Median			99-Percentile		
		S	CCNN-S	MFNN-S	S	CCNN-S	MFNN-S	S	CCNN-S	MFNN-S
Tj-1	Roll(deg)	0.923	0.021	0.033	−0.930	0.003	−0.004	−0.388	0.122	0.154
	Pitch(deg)	0.530	0.037	0.041	0.547	−0.002	0.000	0.711	0.043	0.133
	Heading(deg)	0.358	0.135	0.145	−0.222	0.005	0.002	0.754	0.679	0.741
	North (m)	0.307	0.218	0.277	0.019	−0.002	0.003	0.619	0.412	0.301
	East (m)	0.235	0.107	0.099	0.017	0.000	0.004	0.677	0.309	0.292
	Height (m)	0.445	0.140	0.150	0.027	−0.002	−0.002	2.298	0.203	0.574
Tj-2	Roll (deg)	0.843	0.130	0.160	−0.840	−0.006	0.004	−0.232	0.071	0.224
	Pitch (deg)	0.671	0.102	0.110	0.672	−0.001	0.001	0.971	0.136	0.142
	Heading(deg)	0.302	0.192	0.204	−0.224	0.000	0.000	0.889	0.143	1.265
	North (m)	0.622	0.424	0.440	0.009	0.000	0.005	1.677	0.898	1.445
	East (m)	0.844	0.687	0.766	−0.083	0.000	0.000	0.438	0.395	0.500
	Height (m)	0.327	0.124	0.145	0.033	0.001	0.001	1.697	0.217	0.598
S: Smoother; CCNN-S: CCNN-Smoother; MFNN-S: MFNN-Smoother										

**(c)** Testing scenario-III.										
States		RMS			Median			99-Percentile		
		S	CCNN-S	MFNN-S	S	CCNN-S	MFNN-S	S	CCNN-S	MFNN-S
Tj-1	Roll(deg)	0.925	0.023	0.033	−0.931	0.003	−0.003	−0.384	0.127	0.173
	Pitch(deg)	0.531	0.038	0.038	0.547	−0.002	0.001	0.711	0.044	0.149
	Heading(deg)	0.372	0.179	0.184	−0.222	0.005	0.002	0.755	0.573	0.639
	North (m)	0.318	0.256	0.263	0.019	−0.001	0.002	0.630	0.385	0.309
	East (m)	0.244	0.122	0.114	0.017	0.000	0.004	0.694	0.292	0.294
	Height (m)	0.455	0.134	0.150	0.027	−0.002	−0.001	2.209	0.220	0.656
Tj-2	Roll (deg)	0.852	0.122	0.123	−0.856	−0.005	0.004	−0.299	0.075	0.665
	Pitch (deg)	0.667	0.102	0.105	0.667	−0.001	0.001	0.928	0.128	0.105
	Heading(deg)	0.309	0.201	0.205	−0.224	0.000	0.000	0.995	0.133	1.097
	North (m)	0.481	0.347	0.428	0.013	0.000	0.005	1.702	0.833	1.543
	East (m)	1.043	0.823	1.021	−0.084	0.000	0.001	0.485	0.323	0.535
	Height (m)	0.319	0.114	0.138	0.033	0.001	0.002	1.696	0.233	0.579
S: Smoother; CCNN-S: CCNN-Smoother; MFNN-S: MFNN-Smoother										

**(d)** Testing scenario-IV.										
States		RMS			Median			99-Percentile		
		S	CCNN-S	MFNN-S	S	CCNN-S	MFNN-S	S	CCNN-S	MFNN-S
Tj-1	Roll(deg)	0.932	0.080	0.394	−0.931	0.002	−0.003	−0.369	0.129	0.123
	Pitch(deg)	0.534	0.045	0.047	0.547	−0.002	0.001	0.711	0.048	0.207
	Heading(deg)	0.413	0.103	0.180	−0.222	0.005	0.001	0.753	0.519	0.606
	North (m)	0.367	0.256	0.271	0.019	0.000	0.000	0.618	0.338	0.264
	East (m)	0.273	0.173	0.165	0.016	0.000	0.002	0.673	0.445	0.466
	Height (m)	0.470	0.175	0.164	0.027	−0.001	0.001	2.250	0.227	0.764
Tj-2	Roll (deg)	0.864	0.220	0.271	−0.869	−0.006	0.000	−0.082	0.068	0.073
	Pitch (deg)	0.731	0.163	0.176	0.685	−0.001	0.003	1.784	0.129	0.970
	Heading(deg)	0.315	0.228	0.289	−0.226	0.000	−0.001	0.937	0.127	0.427
	North (m)	0.937	0.526	0.662	0.015	0.000	0.004	1.765	0.779	0.960
	East (m)	1.550	1.322	1.345	−0.089	0.000	0.001	0.450	0.350	0.424
	Height (m)	0.326	0.140	0.187	0.033	0.000	0.002	1.693	0.289	0.714
S: Smoother; CCNN-S: CCNN-Smoother; MFNN-S: MFNN-Smoother										

**Table 5.** The comparison between MFNN and CCNN methodologies.

**ANN**	**Strengths**	**Weaknesses**
**MFNN**	*Easy to implement* *Popular* *Various learning algorithms*	*Time consuming with empirical guessing* *Overtraining issue.* *Difficult to re-train* *Higher adaptability to new data* *Bad for knowledge accumulation*
**CCNN**	*Faster learning process.* *Better generalization* *Grows topology automatically.* *Uses input parameters more effectively.* *Higher adaptability to new data.* *Easy to re-train.* *Good for knowledge accumulation*	*Sensitive to input vectors and requires suitable preprocessing.* *Not popular*
